# Multicenter Epidemiological Study to Assess the Population of CKD Patients in Greece: Results from the PRESTAR Study

**DOI:** 10.1371/journal.pone.0112767

**Published:** 2014-11-18

**Authors:** Konstantinos Sombolos, Demitrios Tsakiris, John Boletis, Demetrios Vlahakos, Kostas C. Siamopoulos, Vassilios Vargemezis, Pavlos Nikolaidis, Christos Iatrou, Eugene Dafnis, Konstantinos Xynos, Christos Argyropoulos

**Affiliations:** 1 Nephrology Clinic, Papanikolaou Hospital, Thessaloniki, Greece; 2 Nephrology Clinic, Papageorgiou Hospital, Thessaloniki, Greece; 3 Nephrology Clinic, Laiko Hospital, Athens, Greece; 4 Nephrology Clinic, ATTIKON University Hospital, Athens, Greece; 5 Nephrology Clinic, Ioannina University Hospital, Ioannina, Greece; 6 Nephrology Clinic, Alexandroupoli University Hospital, Alexandroupoli, Greece; 7 Nephrology Clinic, AHEPA Hospital, Thessaloniki, Greece; 8 Center for Nephrology, G Papadakis, Nikea Hospital, Athens, Greece; 9 Nephrology Clinic, PE.PA.G.N.I, Crete University Hospital, Iraklio, Greece; 10 AbbVie Pharmaceuticals, Chicago, Illinois, United States of America; 11 AbbVie Pharmaceuticals SA, Athens, Greece; University of Sao Paulo Medical School, Brazil

## Abstract

**Background:**

Chronic Kidney Disease (CKD) is a relatively common condition not only associated with increased morbidity and mortality but also fuelling End Stage Renal Disease (ESRD). Among developed nations, Greece has one of the highest ESRD incidence rates, yet there is limited understanding of the epidemiology of earlier stages of CKD.

**Methods:**

Cross-sectional survey of pre-dialysis CKD outpatients in nephrology clinics in the National Health Care system between October 2009 and October 2010. Demographics, cause of CKD, blood pressure, level of renal function, duration of CKD and nephrology care, and specialty of referral physician were collected and analyzed. Different methods for estimating renal function (Cockroft-Gault [CG], CKD-Epi and MDRD) and staging CKD were assessed for agreement.

**Results:**

A total of 1,501 patients in 9 centers were enrolled. Diabetic nephropathy was the most common nephrologist assigned cause of CKD (29.7%). In total, 36.5% of patients had self-referred to the nephrologist; patients with diabetes or serum creatinine above 220 µmol/l (eGFR<40 ml/min/1.73 m^2^) were more likely to have been referred by a physician. Agreement between MDRD and CKD-Epi, but not between CG, the other estimating equations, was excellent. There was substantial heterogeneity with respect to renal diagnoses, referral patterns and blood pressure among participating centers.

**Conclusions:**

In this first epidemiologic assessment of CKD in Greece, we documented delayed referral and high rates of self-referral among patients with CKD. eGFR reporting, currently offered by a limited number of laboratories, may facilitate detection of CKD at an earlier, more treatable stage.

## Introduction

Chronic Kidney Disease (CKD) is a relatively common condition associated with increased morbidity and mortality, mainly due to cardiovascular causes [1], [2]. The increasing prevalence of risk factors for CKD such as obesity, diabetes and hypertension appears to fuel an emergent CKD epidemic on a global scale [3]. Contrary to patients with End Stage Renal Disease (ESRD), the care of patients with pre-dialysis CKD is primarily being overseen by general medicine, primary care physicians (PCPs), with specialist (nephrologist) input provided for patients with advanced stages of CKD [4]–[6]. These practice patterns translate to substantial missed opportunities to optimize care of patients with CKD in terms of disease education, selection of dialysis modality, pre-emptive transplantation, and implementing plans for the timely creation and maturation of dialysis access [7]–[9]. Furthermore, referral and [10]–[12] treatment in nephrology clinics has been shown to decrease the rate of progression of CKD and optimize the treatment of CKD complications [13].

In spite of these advantages, our understanding of how patients are referred for pre-dialysis nephrology consultation is limited, especially in settings in which formal partnerships between PCPs and nephrologists and referral recommendations for patients with CKD are not in place [14]. Furthermore, such information is rarely available on a country-wide basis, limiting the possibility of linking pre-dialysis practices to dialysis treatment pattern and outcomes in national registries. This is particularly important in settings characterized by high incidence of ESRD, given the toll the disease exacts on patients, caregivers and healthcare systems. Greece has one of the highest ESRD incidence rates [15] among industrialized nations, yet there is limited understanding of the epidemiology of earlier stages of CKD at the national level. In this cross-sectional, multicenter assessment, we describe the patient characteristics, causes of CKD and distribution of renal function in outpatients of nephrology clinics in the Greek National Healthcare System. Additionally, we characterize the referral patterns in relation to diagnosis of renal disease and the level of renal function, and describe center specific variations in these parameters. Finally, we examine the relative performance of different equations for estimated Glomerular Filtration Rate (eGFR) in the Greek CKD population.

## Subjects and Methods

### Design and Participants

This is a multicentre, observational, cross-sectional epidemiological study conducted in 9 outpatient Nephrology Clinics of the National Health System from across the different regions of Greece from October 2009 to October 2010. Centers were selected for participation based on previous workload and catchment area that included both urban and rural segments of the population. The sample size for the study was selected to ensure that the percentage of CKD 3–5 patients will be estimated with a degree of error of 2.3% when the expected percentage is 70%. Patients were eligible to participate in the study if they were older than 18 years, able to give informed consent, established patients of the clinic, not currently receiving renal replacement therapy. Patients with acute kidney injury/acute renal failure and patients with neoplasms or any other serious disease with projected life expectancy of less than 12 months were excluded from the study to ensure that only medically stable outpatients could be observed. During the course of the study the following data were collected from consecutive patients: demographics, cause of CKD assigned by the treating consultant nephrologist, day of first diagnosis of CKD and first nephrology visit, intervening period between first diagnosis and the study visit and between first nephrology visit and the study visit, specialty of referring physician, blood pressure, weight and height and laboratory values of blood urea nitrogen, creatinine and PTH available for physician review during the study visit. Field collected data were used to derive body mass index (BMI) and different estimates of renal function (estimated creatinine clearance by Cockroft-Gault (CG), CG normalized to Body Surface Area (CG-BSA) and estimated glomerular filtration rate (eGFR) according to the CKD-Epi and the MDRD equations (the latter modified to use standardized creatinine values which was almost invariably used by Greek clinical laboratories during the study). For this study, renal function was classified by CKD stage according to the numerical cutoffs in the NKF classification system for estimates normalized to BSA (MDRD, CKD-Epi, CG-BSA) as well as the CG equation. This study was conducted in accordance with a pre-specified protocol and applicable local regulations and guidelines. The protocol was prospectively registered with the National Medicines Organization (registration code EE 25/01-09/09) and approved by the Institutional Review Board of each participating center (Hospital Scientific Committee of Papanikolaou General Hospital Decision of the 3^rd^ Meeting/31-3-2009, Hospital Scientific Committee of Papageorgiou General Hospital Decision of the 131th Meeting/25-05-2009, Hospital Scientific Committee of Laiko Hospital Decision: 114/3-6-09, Hospital Scientific Committee of Attikon University Hospital Decision of the 2^nd^ Meeting/16-03-2009, Hospital Scientific Committee of University Hospital of Ioannina Decision: 196/30-6-2009, Hospital Scientific Committee of University Hospital of Alexandroupoli Decision: 378/9-06-2009, Hospital Scientific Committee of AHEPA Hospital Decision: 257/6-5-2009, Hospital Scientific Committee of Crete University Hospital Decision: 3564/5-5-2009), and each participant provided written informed consent before patient enrollment.

### Statistical Methods

Descriptive statistical methods were used to summarize the distribution of all variables: for continuous variables the mean and the standard deviation, and for categorical variables the frequencies of responses at each level were reported. Unadjusted assessments were carried out with the Kruskal Wallis test (continuous responses) and the chi-square test (discrete responses). The pair-wise agreement between estimating equations for renal function was examined by means of Bland Altman plots [16]. We assessed agreement between CKD stages based on all other estimating equations compared to the CKD-Epi with the Spearman correlation coefficient. Regression methods were used to examine the relationship between renal function at the time of the study visit and other covariates, using when appropriate, penalized splines for semi-parametric modeling [17]. Center wise comparisons were carried out by random effect models and portrayed as forest plots, while model heterogeneity was assessed by means of the I^2^ statistic. All analyses were performed with SAS v9.1 (table generation) and R v2.12.1 (meta-analyses and figures).

## Results

### Patient Characteristics and Comparison of Renal Function Estimating Equations

The study included 1501 patients from 9 outpatient nephrology clinics, with a range of 80–300 patients per clinic. Patients on average were older (mean age 66.2±14.6 years, median: 69.6 years), predominantly male (54. 9%), slightly overweight (median BMI: 27.8 kg/m^2^). The three most commonly assigned causes of CKD were: diabetic nephropathy (29.7%), hypertensive vascular disease (25.3%) and glomerular diseases (16.3%). The average time interval since the initial diagnosis of CKD was 4.4±5.5 years, while the average time interval since the initial nephrology consultation was 3.0±3.7 years. Other patient characteristics are shown in Table 1.

**Table 1:** Patient characteristics.

**Age** (yrs)	66.2 (14.6)
**Gender** (Male)	824 (55%)
**Time since initial diagnosis of renal disease** (yrs)	4.4±5.5
**Time since initial nephrology consultation** (yrs)	3.0±3.7
**Systolic Blood Pressure** (mmHg)	137.1±17.8
**Diastolic Blood Pressure** (mmHg)	80.5±10.4
**Cause of CKD**	
Diabetic Nephropathy	445 (29.7)
Hypertensive Vascular Disease	380 (25.3)
Glomerular Disease	244 (16.3)
Interstitial Nephropathy	75 (5.0)
Solitary kidney post nephrectomy	61 (4.1)
Arterial Hypertension – Congestive Heart Failure	56 (3.7)
Obstructive Nephropathy	38 (2.5)
Polycystic Kidney Disease	35 (2.3)
Autoimmune Disease	29 (1.9)
Nephrolithiasis	28 (1.9)
Dysplastic/Hypoplastic Disease	21 (1.4)
Malignancy	12 (0.8)
Post infectious	7 (0.5)
Drug Related	4 (0.3)
Others	115 (7.7)
**Weight** (kg)	77.9±15.5
**Height** (cm)	164.7±9.4
**Body Mass Index** (BMI, kg/m^2^)	28.8±5.5
**Body Surface Area** (BSA, m^2^)	1.84±0.20
**Blood Urea Nitrogen** (mmol/l)	80.9±45.8
**Serum creatinine** (µmol/l)	176.8±97.2
eGFR (by CKD-Epi, ml/min/1.73 m^2^)	40.9±23.9

Data are given as Mean ±SD or n (%) unless stated otherwise.

**Figure 1:**
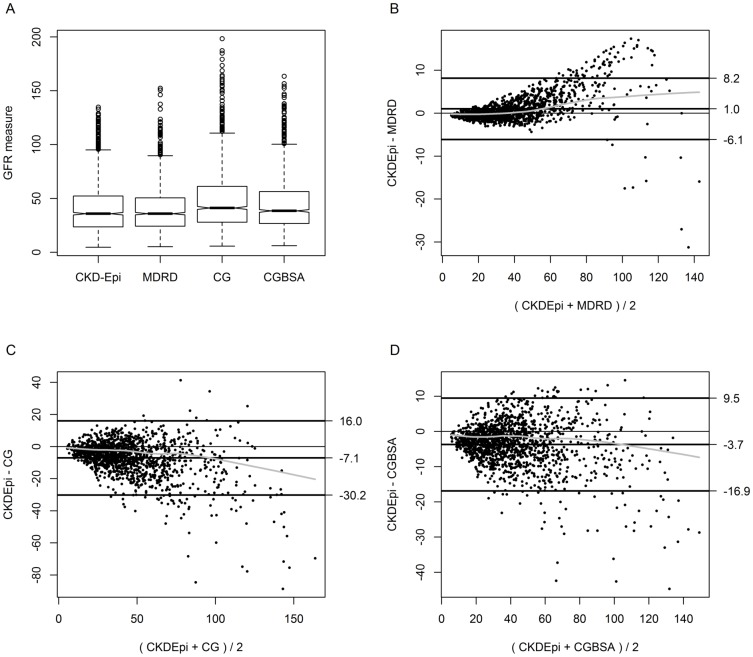
Estimating equations for renal function in the PreSTAR study (A) Box whisker plots of individual estimates based on the CKD Epi (CKD-Epi), MDRD, Cockroft Gault (CG) and Cockroft Gault normalized to Body Surface Area (CGBSA). The thick horizontal line is the median estimate, the bottom and top of each box are the 25^th^ and 75^th^ percentiles, the thin horizontal lines are the most extreme data points within 1.5 times the interquartile range, while the outliers are shown as circles. (B–D) Bland Altman (BA) plot of the MDRD (B), CG(C) and CGBSA against CKD-Epi (D). Each BA plot shows the difference between the two levels of renal function (y-axis) against their average (x-axis) for each patient. The three thick black lines demarcate the bias and the upper and lower limits of agreement, while the thin horizontal line is the zero bias line. In each plot, the gray line is a non-parametric estimate of the constancy of the bias across the range of possible values.

**Figure 2:**
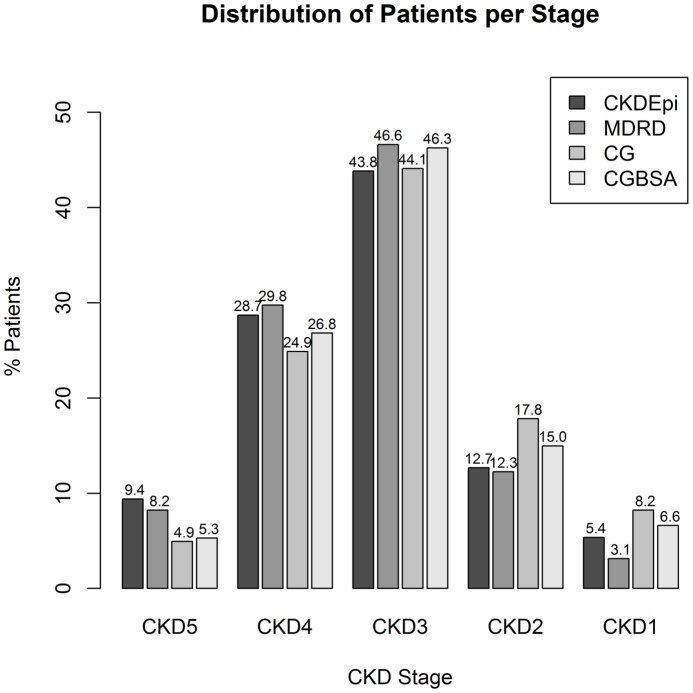
Distribution of stages of CKD in PRESTAR according to different estimating equations, CKD-Epi, MDRD, Cockroft Gault (CG), CG normalized to Body Surface Area (CGBSA).

Average eGFR (by CKD-Epi) was 40.9±23.9 ml/min/1.73 m^2^, similar to the MDRD estimate (39.9±22.1 ml/min/1.73 m^2^). Estimating renal function by CG, or GC normalized to BSA (CGBSA) resulted in higher numerical values (Figure 1A) than either MDRD or CKD-Epi. Agreement was best between CKD-Epi and MDRD (Figure 1B, average bias 1 ml/min/1.73 m^2^, although MDRD yielded lower estimates for values>60 ml/min/1.73 m^2^) and worse between CG and CKD-Epi (Figure 1C); normalization of CG to BSA (CGBSA) improved agreement (Figure 1D, average bias −3.7 ml/min/1.73 m^2^). Approximately 44% of all patients would be classified as having CKD Stage 3 irrespective of the estimating equation used, while CG and CGBSA classification led to higher prevalence estimates of stages 1–2 in the study population (Figure 2). The correlation between CKD stages based on different estimating equations was highest between MDRD and CKD-Epi (Spearman's ρ 0.96) and lowest between MDRD and CG (Spearman's ρ 0.82).

### Renal Function, Cause of CKD and Referral Patterns

The level of renal function (eGFR) in nephrology outpatients appeared to differ according to the renal diagnosis, with diabetic patients having lower eGFR, while patients with glomerular disease having higher eGFR (Table 2). Overall, 63.2% (948/1501) of all CKD patients had been referred to a nephrologist by a physician, most commonly an internist (48.0% of referred patients), a diabetologist or a cardiologist in the overall population and across the different CKD stages (Table 3).

**Table 2:** Renal function (eGFR by CKD-Epi in ml/min/1.73 m^2^) according to presence or absence of renal diagnosis.

Renal Diagnosis	Present	Absent	P-value
Diabetic Nephropathy	34±18	45±28	<0.001
Hypertensive Vascular Disease	38±19	43±27	0.089
Glomerular Disease	52±29	40±24	<0.001
Interstitial Nephropathy	42±26	42±26	0.830
Polycystic Kidney Disease	48±24	42±26	0.047

**Table 3:** Specialty of physicians referring patients to Greek National Health System Outpatient Nephrology Clinics.

	Stage 1	Stage 2	Stage 3	Stage 4	Stage 5	All
Referring Specialty	51 (100%)	106 (100%)	425 (100%)	265 (100%)	101 (100%)	948 (100%)
Internal Medicine	26 (51.0)	47 (44.3)	194 (45.6)	138 (52.1)	50 (49.5)	455 (48.0)
Diabetology	1 (2.0)	12 (11.3)	43 (10.1)	30 (11.3)	10 (9.9)	96 (10.1)
Cardiology	0 (0.0)	2 (1.9%)	50 (11.8)	23 (8.7)	9 (8.9)	84 (8.9)
Urology	4 (7.8)	9 (8.5)	31 (7.3)	17 (6.4)	4 (4.0)	65 (6.9)
Endocrinology	1 (2.0)	9 (8.5)	26 (6.1)	15 (5.7)	7 (6.9)	58 (6.1)
General Surgery	2 (3.9)	4 (3.8)	18 (4.2)	8 (3.0)	1 (1.0)	33 (3.5)
Rheumatology	2 (3.9)	5 (4.7)	10 (2.4)	6 (2.3)	3 (3.0)	26 (2.7)
Hematology	3 (5.9)	3 (2.8)	12 (2.8)	2 (0.8)	2 (2.0)	22 (2.3)
GP/Family Medicine	0 (0.0)	2 (1.9)	5 (1.2)	7 (2.6)	2 (2.0)	16 (1.7)
Nephrology	1 (2.0)	3 (2.8)	9 (2.1)	2 (0.8)	1 (1.0)	16 (1.7)
Pulmonary Medicine	0 (0.0)	3 (2.8)	5 (1.2)	4 (1.5)	1 (1.0)	13 (1.4)
Neurology	0 (0.0)	1 (0.9)	3 (0.7)	2 (0.8)	2 (2.0)	8 (0.8)
Vascular Surgery	0 (0.0)	1 (0.9)	3 (0.7)	3 (1.1)	1 (1.0)	8 (0.8)
All others	11 (21.5)	5 (4.7)	16 (3.8)	8 (3.0)	8 (7.9)	48 (4.8)

The number of patients (N = 948) in this table differs from the total number of patients in the study (N = 1501), because N = 1501–948 = 553 patients were self-referrals. Data are given as n(%).

**Figure 3:**
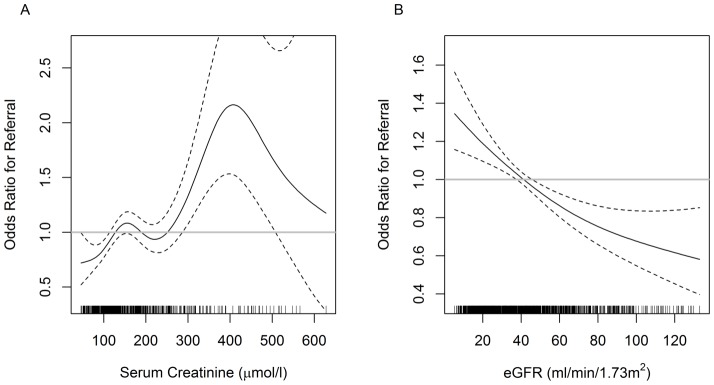
Adjusted odds of a patient having been referred to a nephrologist by a specialist in relation to serum creatinine (A) and eGFR (CKD-Epi), (B) during the study visit. Models were adjusted for age, gender, race, presence of diabetic renal disease, center, systolic and diastolic blood pressure and time the patient had been under nephrology care. Solid black line: estimated adjusted odds ratio, dashed black lines: associated pointwise 95% confidence internal, gray horizontal line: corresponds to an odds ratio of one.

The likelihood of a patient being referred to a nephrologist were assessed as a function of serum creatinine concentration (Figure 3A) and eGFR (Figure 3B) adjusted for the time the patient had spent under nephrology care. The odds ratio of a referral increased as serum creatinine increased above 88.4 µmol/l (1 mg/dl) and peaked around 150 µmol/l and remained relatively stable until 220 µmol/l, to increase thereafter. Stated in other terms, the patients were much more likely to be referred for a creatinine concentration exceeding 220 µmol/l (∼2.5 mg/dl) than at lower creatinine values. Viewed as a function of eGFR, the odds of a nephrology referral were a monotonic (decreasing) function of the estimated GFR, so that patients with eGFR<40 ml/min/1.73 m^2^ were more likely to have been referred to a specialist than to have self-referred.

In unadjusted analyses examining renal diagnosis and likelihood of referral, patients with diabetes were more likely to had been referred to a nephrologist by another physician (Odds Ratio 1.82, 95% CI 1.61–2.06, p<0.001), while patients with glomerular and interstitial disease were less likely to have been referred (Table 4). In analyses adjusting for age, gender, SBP, DBP, eGFR (CKD-Epi), center, and time spent under nephrology care, patients with diabetes were more likely to have been referred to a nephrologist (OR: 1.73, 95% CI: 1.25–2.38, p = 0.036), while patients with polycystic kidney disease were less likely to have been referred (OR: 0.69, 95% CI: 0.48–0.97, p = 0.032). Age was not independently associated with lower odds of nephrology referral (p>0.10).

**Table 4:** Likelihood of a physician referral to a nephrologist by renal diagnosis.

	Unadjusted Analyses			Adjusted Analyses		
Renal Diagnosis	OR	95% CI	P	OR	95% CI	P
Diabetic Nephropathy	1.82	1.43–2.32	<0.001	1.73	1.25–2.39	<0.001
Hypertensive Vascular Disease	0.82	0.65–1.05	0.12	0.40	0.16–1.00	0.051
Glomerular Disease	0.46	0.35–0.61	<0.001	0.73	0.50–1.07	0.10
Interstitial Nephropathy	0.57	0.36–0.91	0.02	0.97	0.52–1.81	0.92
Polycystic Kidney Disease	0.60	0.31–1.18	0.14	0.69	0.49–0.97	0.032

### Center level variations

**Figure 4:**
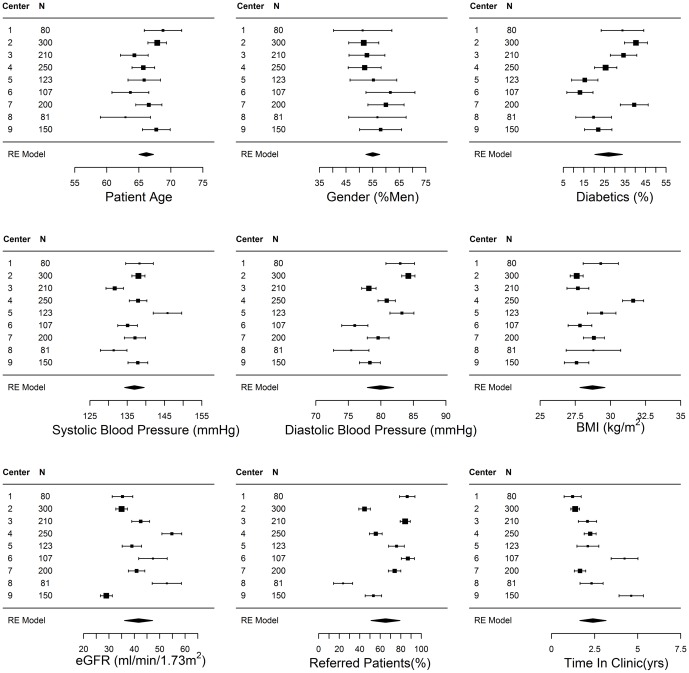
Center wise variations in patient demographics, blood pressure, renal function, diabetes and percentage of referred patients (Could number of patients in each center be added as a footnote?).

Large center-wise variations and heterogeneity were observed in a number of characteristics of the outpatients attending nephrology clinics (Figure 4): percentage of patients with diabetes (I^2^: 89.19%, p<0.001), CKD-Epi eGFR (I^2^: 95.74%, p<0.001) SBP (I^2^: 88.58%, p<0.001), DBP (I^2^: 93.56%, p<0.001), BMI (I^2^: 90.4%, p<0.001), percentage of patients referred by a physician (I^2^: 97.62%, p<0.001), and time under care in the nephrology clinic(I^2^: 96.16%, p<0.001).

## Discussion

The purpose of this research was to report for the first time the characteristics of the outpatients attending the nephrology clinics of the Greek National Health System (GHNS). We found that a large proportion of patients had referred themselves to nephrologists, with the likelihood of self-referral increasing with higher eGFR levels. Diabetic patients were more likely to have been referred by a physician, even after adjusting for eGFR and other factors. There appears to be some heterogeneity at the center level with respect to the proportion of diabetic patients and self-referrals and the level of blood pressure.

The findings of this report need to be interpreted in light of several factors operating in the Greek healthcare system: a) patients have direct access to laboratory results, irrespective of the public or private ownership of the laboratory, b) lack of automatic eGFR reporting by the laboratories, c) lack of consensus guidelines for referrals to specialists, and finally d) extremely low barriers limiting patient access to specialty care in the outpatient clinics of the GNHS.

Direct patient access to laboratory results, along with their reference range, probably explains the large percentage of self-referrals in this study. Under this scenario, it is very likely that small changes in serum creatinine levels or dipstick positive albuminuria are interpreted by patients as indicating a potential problem with their kidneys. This in turn will prompt them to seek specialty evaluation in the GHNS, which by design does not have a gatekeeper mechanism to restrict access and only commissions a small fee for specialty evaluation. Hence many patients with relatively preserved renal function, but some form of renal pathology, will end up being evaluated by a nephrologist. From this perspective, direct patient access to creatinine test results may provide a less costly alternative to screening programs [9], [18] for the identification of patients who may benefit from specialty care [19].

On the other hand, patients referred to a nephrologist by another physician had lower levels of eGFR, implying that either renal disease is under-recognized or some form of filtering of patients by referring physicians is taking place. Such a filtering has been observed in other settings, in which only a small minority (27%) of patients with CKD would be identified as such by their primary providers [20], while large increases in serum creatinine (36%) are used as triggers for patient referral. As very few laboratories currently report eGFR in Greece, we postulate that recognition of renal impairment by non-nephrologists is largely based on serum creatinine concentration, which is an insensitive marker of renal dysfunction [21]. This hypothesis is supported by our finding that the odds of referral as a function of serum creatinine level exceeds parity only when serum creatinine increases above the upper limit of normal (∼110 µmol/l) but substantially increases only when creatinine is higher than 220 µmol/l (∼2.5 mg/dl), or approximately two times the upper limit of normal. This level, clearly understood to warrant specialty evaluation in the pre-eGFR days [22], coincides with the putative “point of no return” of many renal diseases [23], [24] suggesting that some patients may in fact receive sub-optimal care due to such filtering. In contrast, self-referring patients do not seem to apply such filtering, seeking specialty evaluation even when creatinine has increased just above the range of normal values reported by the laboratory. This finding is no different from previous reports showing that non nephrologists will refer late, i.e. after creatinine is higher than 177 µmol/l(∼2.0 mg/dl) [25], a pattern which may be due to limited awareness of the need for early specialty evaluation and care [25]–[31]. Alternatively such late referrals can be due to perceptions of the nephrologist role as one of transitioning the patient with advanced CKD towards a plan for ESRD management when eGFR declines below 30 ml/min/1.73 m^2^ [32]. In that regards, automatic eGFR reporting, which has been shown to aid the identification of subtle renal impairment [33]–[35], increase the prescription rate of nephro-protective ACEis/ARBs [33], [36], [37] and the probability of specialty referral [38], may be viewed as an important tool for the management of CKD patients by primary care practitioners. On the other hand, eGFR reporting may increase the number of inappropriate evaluations and the nephrologist workload [39], as patients are seen at higher levels of eGFR. Nevertheless, recent evaluations have shown that although consults increase [36], [40], the proportion of inappropriate consults does not invariably go up [40], [41], the reported eGFR does not influence the rate of consults among patients without CKD, and the additional workload is modest (23 additional consults per nephrologist per year) [42].

Early nephrology referral has been associated with slower disease progression and a 45% reduction in the risk of death [43], and is thus an important aspect of a comprehensive CKD population health care program. Furthermore, patients referred late have inferior control of risk factors for CKD progression, CKD complications, uremic cardiomyopathy and worse patient survival [44], [45] when they reach dialysis [7], [46]. On the other hand, specialty referral has been shown to lead to higher rates of prescriptions for ACEis/ARBs [47], NSAID avoidance [48], stabilization or improvement in renal function decline CKD [12], [43], [49] and improved survival among patients with consistent nephrology follow-up [50], [51].

Since eGFR reporting may be a valuable component for the optimization of pre-dialysis CKD care and it is currently not implemented on a large scale in the Greek health care system, we explored the utility of different estimating equations for either GFR or creatinine clearance. Assuming the CKD-Epi equation as the emerging gold standard [52], [53], our analyses highlight potential pitfalls of the other methods including the imprecision of the MDRD at higher levels of eGFR, the large bias of the CG (moderated somewhat by scaling the result to BSA) and the potential for misclassification at earlier CKD stages. This is particularly important from the perspective of public health expenditure in Greece, as therapies targeting complications of CKD stages 3–5 are currently fully reimbursed without any patient copayment (currently standing at 25% of the price of non CKD related therapies) Hence accurate staging of CKD is important for both early identification and public health budget optimization, goals that can be attained by widespread adoption of the CKD-Epi equation in the GNHS.

In this report, we also observed center variations in a number of characteristics relating to diagnosis, pathway to specialty evaluation, and blood pressure levels of CKD patients under nephrology care. Since the participating centers in this study cover both rural and urban segments of the Greek population, it is possible that some of these differences directly reflect some heterogeneity in the distribution of risk factors for CKD, local practice patterns of referring physicians and nephrologists and models of cooperation between them. Nevertheless, the fact that such differences do exist, suggest a missed opportunity for standardizing care at a national level by e.g. CKD diagnosis and treatment educational programs aiming at non-specialists and promotion and adoption of guidelines specifying thresholds for appropriate referral and blood pressure targets.

The findings and interpretations in this report should be interpreted in light of certain limitations. First, the cross-sectional design of the study precludes drawing conclusions about the rate of renal function decline over time among study participants. Second, we did not have access to referring physicians' records so we cannot explain the apparent channeling bias in referring patients with diabetes to a nephrologist. This pattern may reflect the limited understanding of the subtle manifestations of a wide spectrum of renal pathology by non-specialists, the sensitization of non-nephrologists to the renal complications of diabetes [54], or possibly a skewed view by non-specialists about the benefits of nephrologist co-management [13], [49], [55]–[57]. A study of referring physicians could help us understand these referral patterns, suggesting one possible way for filling this knowledge gap. Third, even though the MDRD and CKD-Epi equations have been evaluated in a number of different populations and cohorts, no direct validation exists for the Greek population, so that the comparison between these equations should not be viewed as one of a method against a gold standard. Finally our study was conducted before the 2012 KDIGO classification of CKD stages along the two dimensions of eGFR and albuminuria categories, and thus we did not collect data about abnormal urinary biomarkers (proteinuria, albuminuria or even hematuria) in our patients. Hence, we cannot exclude the likelihood that such abnormalities drive nephrology referrals at higher levels of eGFR especially for patients with glomerular disease who are more likely to manifest proteinuria and/or hematuria.

In summary, we have undertaken the first national cross-sectional evaluation of non-dialysis dependent CKD patients in outpatient nephrology clinics of the Greek National Health System. We found that many patients appear to be referred late by physicians, while self-referred patients consult a nephrologist at a higher level of renal function possibly due to direct access to test results. To reduce the burden of ESRD in Greece, with the 8^th^ highest incidence rate in the world [15], which during the current financial crisis bears the cost of providing unfunded dialysis services to a large number of uninsured individuals including illegal immigrants future initiatives should focus on the adoption of eGFR reporting in order to facilitate early detection, appropriate confirmatory testing, and prescription of reno-protective medications in order to reduce the progression to dialysis dependency.
